# Hijacking erythropoietin: A tumour‐intrinsic strategy to escape immune surveillance

**DOI:** 10.1002/ctm2.70427

**Published:** 2025-07-27

**Authors:** David Kung‐Chun Chiu, Edgar G Engleman

**Affiliations:** ^1^ Department of Pathology Stanford University Stanford California USA; ^2^ Stanford Cancer Institute Stanford University Palo Alto California USA

1

An increasingly recognised framework in cancer immunology classifies tumours into T‐cell‐inflamed and non‐inflamed phenotypes, based on the degree of immune cell infiltration, particularly cytotoxic CD8⁺ T cells.^1^ Early studies demonstrated that CD8⁺ T‐cell infiltration is one of the most informative immune features associated with a favourable prognosis.^2^ Building on this insight, prognostic tools such as the Immunoscore were developed to quantify the density and spatial distribution of CD3⁺ and CD8⁺ T cells within the tumour core and invasive margin. This approach has shown that CD8⁺ T‐cell infiltration can serve as a robust, immune‐based classifier that outperforms traditional TNM staging in predicting clinical outcomes.^3^ The introduction of immune checkpoint inhibitors (ICI) targeting the PD‐1/PD‐L1 axis has transformed cancer therapy. However, response rates remain limited across many tumour types. One hypothesis is that effective responses to ICI require pre‐existing anti‐tumour T‐cell immunity. Indeed, pioneering studies in melanoma demonstrated that patients with higher CD8⁺ T‐cell densities at the invasive tumour margin were significantly more likely to respond to PD‐1 blockade. Supporting this concept, a Phase II clinical trial in colorectal cancer showed that patients with a low Immunoscore did not benefit from PD‐L1 blockade, whereas those with a high Immunoscore experienced a 65% reduction in disease progression risk.^4^ These findings have led to the now widely accepted view that non‐inflamed tumours are a major cause of resistance to ICI. This realisation has catalysed two central questions in the field: What determines whether a tumour is non‐inflamed? And how can we reprogram non‐inflamed tumours into an inflamed, immunologically active state?

To investigate these questions, our laboratory utilised a collection of autochthonous mouse models of hepatocellular carcinoma (HCC), each induced by different driver mutations and characterised by a defined T‐cell‐inflamed or non‐inflamed immune phenotype.^5^ Compared to conventional transplantable or cell line‐based models, these autochthonous models more accurately recapitulate the complex features of human tumours, including their immune tumour microenvironment and responsiveness to ICI. Through direct comparison of T‐cell‐inflamed and non‐inflamed HCC tumours, we identified that non‐inflamed tumours exhibited significantly elevated levels of erythropoietin (EPO). Forced expression of EPO in the tumour cells of T‐cell‐inflamed tumours was sufficient to convert the tumours into a non‐inflamed phenotype, marked by a significant reduction in CD8⁺ T‐cell infiltration. Conversely, genetic ablation of EPO in non‐inflamed tumours restored T‐cell infiltration, effectively reprogramming the tumours into an inflamed, immune‐responsive state. Mechanistically, we observed that macrophages are the predominant immune cell type expressing the EPO receptor (EPOR) in non‐inflamed tumours. Selective deletion of EPOR in these macrophages triggered a robust antitumour CD8⁺ T‐cell response, indicating that EPO/EPOR signalling suppresses macrophage activation and their function as proinflammatory antigen‐presenting cells. These findings suggest that tumour‐derived EPO acts as an immunosuppressive switch, maintaining macrophages in a regulatory, non‐stimulatory state. Interrupting this pathway reprograms the tumour immune microenvironment, facilitating CD8⁺ T‐cell recruitment and converting non‐inflamed tumours into inflamed, ICI‐responsive tumours.^6^

EPO is a protein hormone known mainly for its critical role in stimulating red blood cell production. Following FDA approval of the first recombinant form of EPO (rEPO) in 1989, rEPO became widely used to treat anaemia, particularly in patients with chronic kidney disease and cancer. However, studies in patients with a range of cancers later showed that treatment with rEPO worsened outcomes, including tumour progression and decreased survival.^7^ These concerns led the FDA to issue a black box warning, cautioning against the use of all forms of rEPO in cancer patients. In search of the explanation for these observations, researchers focused primarily on the direct effects of EPO on cancer cells and failed to consider the possibility that it modulates the immune tumour microenvironment. Subsequently, EPO signalling in macrophages was shown to be essential for the phagocytosis of apoptotic cells, a process known to promote immune tolerance.^8^ In addition, mice with macrophage‐specific deletion of EPOR were shown to develop lupus‐like autoimmune symptoms,^8^ suggesting a key role for this pathway in maintaining immune homeostasis. Therefore, while we were initially surprised to discover that EPO is preferentially secreted by tumour cells in noninflamed HCC, it also seemed logical that tumour cells may hijack this underappreciated pathway as a means of evading immune surveillance. What truly startled us was that EPO secreted by HCC not only activates EPOR signalling in macrophages, but that this pathway also plays a central role in governing the distinction between T‐cell‐inflamed and non‐inflamed tumours in our preclinical HCC models.

**FIGURE 1:**
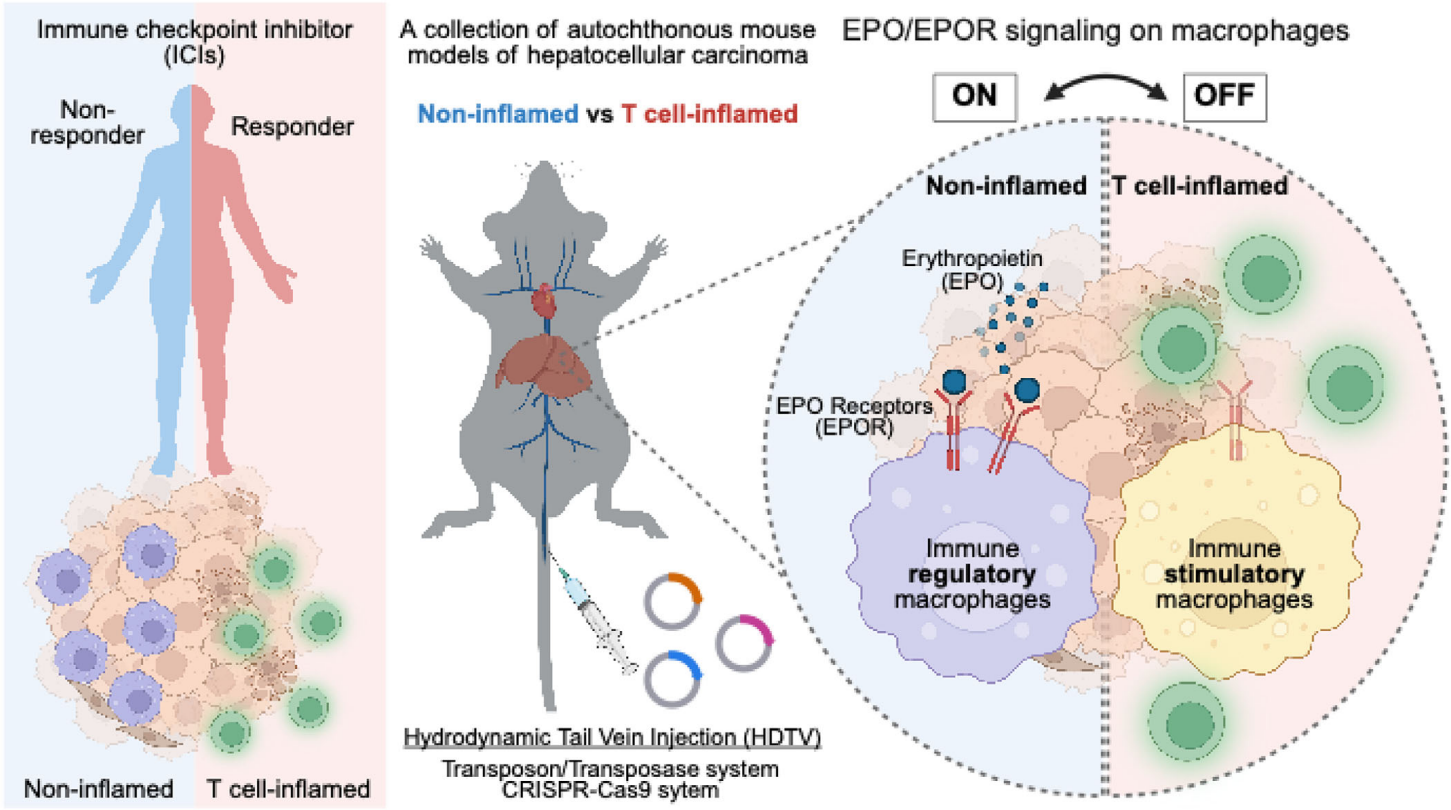
Spontaneous mouse tumour models illuminate EPO/EPOR‐driven immunosuppression in non‐Inflamed liver cancer. Most cancer patients harbour non‐inflamed tumours, which are resistant to immune checkpoint blockade therapy. To explore why certain tumours fail to elicit a robust adaptive immune response, we established a series of spontaneous tumour models characterised by either a T‐cell‐inflamed or non‐inflamed immune microenvironment. Comparative analysis revealed that non‐inflamed tumours secrete high levels of erythropoietin (EPO), and that EPO/EPOR signalling in macrophages functions as an immunosuppressive switch, promoting the development of non‐inflamed tumours.

Our findings^6^ likely explain why rEPO accelerates disease progression in cancer patients. More importantly, our study indicates that targeting EPO/EPOR signalling may reprogram non‐inflamed tumours into an inflamed, immunologically active state. Several critical questions remain to be addressed as we move forward. First, although the EPO/EPOR signalling pathway is highly conserved between mice and humans, and we have observed both EPOR‐expressing macrophages and EPO production in human HCC, it remains unclear whether human HCC also relies on this pathway to evade immunosurveillance. Second, while hepatocytes are among the few cell types known to naturally produce EPO, it is not yet known whether this immunosuppressive mechanism is unique to liver cancer or extends to other tumour types. Could this pathway also be active in non‐HCC tumours or in non‐HCC tumours that metastasise to the liver, where the hepatic environment may facilitate EPO production and immune escape? Finally, although EPOR is a druggable target, a key concern in targeting this pathway therapeutically is the potential for inducing anaemia, given the central role of EPO in erythropoiesis. However, with the advancement of technologies such as nanoparticles, bispecific antibodies, and antibody‐drug conjugates, it may be possible to achieve selective inhibition of EPO/EPOR signalling within the tumour microenvironment, particularly in tumour‐associated macrophages, while sparing red blood cell production. We believe these insights position EPO/EPOR signalling as a tractable target to unlock responses to ICI in tumours previously deemed immunologically silent and therefore resistant to such therapy (Figure 1).
